# Management of Ileal Pouch Cancer in Patients With Inflammatory Bowel Disease

**DOI:** 10.1016/j.gastha.2025.100824

**Published:** 2025-10-01

**Authors:** Shanshan Wang, Huaibin Mabel Ko, Dana J. Lukin, Ellen Scherl, Ravi Kiran, Bo Shen

**Affiliations:** 1 The Global Center for Integrated Colorectal Surgery and IBD Interventional Endoscopy, Columbia University Irving Medical Center/New York Presbyterian, New York, New York; 2 Department of Pathology and Cell Biology, Columbia University Irving Medical Center/New York Presbyterian, New York, New York; 3 Jill Roberts Center for Inflammatory Bowel Disease, Gastroenterology and Hepatology, Department of Medicine, New York Presbyterian/Weill Cornell Medical Center, New York, New York

**Keywords:** cancer, pouch, surveillance, survival, ulcerative colitis

## Abstract

**Background and aims:**

Ileal pouch cancer in patients with inflammatory bowel disease, though rare, represents a significant concern due to the high mortality. While prior studies emphasized incidence and risk factors, this study evaluates the current management and prognosis of pouch cancer following ileal pouch-anal anastomosis.

**Methods:**

Consecutive patients with pouch cancer were identified from a prospectively maintained database (2019–2025). Patients with familial adenomatous polyposis and precancerous conditions were excluded. Demographic, clinical, endoscopic, and histologic data were collected. Primary outcomes were disease recurrence/progression and oncological survival.

**Results:**

Of 699 patients, 10 developed pouch cancer (incidence: 0.5 per 100 person-years). Lesions were located at the cuff (n = 3), anal transition zone (n = 3), pouch body (n = 2), prepouch ileum (n = 1), and both anal transition zone and pouch body (n = 1). Two patients presented with distant metastases without endoscopically visible lesions in the pouch. The histological types were adenocarcinoma (n = 5), squamous cell carcinoma (n = 3), non-Hodgkin lymphoma (n = 1), and neuroendocrine tumor (n = 1). The diagnosis was established through endoscopic biopsies (n = 2), surgical resection (n = 6; three with prior endoscopically diagnosed dysplasia), and cross-sectional imaging (n = 2). Seven patients had localized disease, allowing curative treatment. Treatments included surgery (n = 5), chemoradiotherapy (n = 6), and lanreotide for neuroendocrine tumor. Over a median follow-up of 2.8 years (interquartile range: 1.5–5.2), recurrence or disease progression occurred in three patients (30%), and one patient (10%) died from pouch cancer, with a 5-year cumulative survival of 88.9%.

**Conclusion:**

Adenocarcinoma remains the most common histological type; however, nonadenocarcinoma, especially the squamous cell carcinoma, has been increasingly recognized. Endoscopic surveillance was helpful in detecting precancerous lesions.

## Introduction

Total proctocolectomy (TPC) with ileal pouch-anal anastomosis (IPAA) is the preferred surgical modality for patients with medically refractory ulcerative colitis (UC) or colitis-associated neoplasia (CAN). Compared to colectomy with end ileostomy, IPAA significantly improves the patient’s quality of life.^1^ While colectomy significantly reduces cancer risk, it does not eliminate it. The reported prevalence of ileal pouch cancer for patients with UC ranges from 0.12% to 6.7% in different studies.^2^, ^3^, ^4^, ^5^, ^6^, ^7^, ^8^, ^9^ Although rare, pouch cancer is difficult to detect and is associated with a poor prognosis. The low incidence of pouch cancers has limited the ability to identify consistent and reliable risk factors. To date, the only well-established risk factor for pouch neoplasia is a precolectomy diagnosis of CAN,^4^^,^^10^ while other possible risk factors include primary sclerosing cholangitis (PSC), chronic pouchitis, Crohn’s disease (CD) of the pouch, chronic cuffitis, and family history of colorectal cancer (CRC). Emerging evidence in histopathology suggests CAN follows distinct carcinogenetic pathways compared to sporadic adenomas, characterized by more DNA copy number aberrations and mutations in TP53, FBXW7, APC, and PIK3CA, potentially leading to a faster progression toward cancer.^11^^,^^12^

The prior largest cohort study was published in 2014, including 15 cases of pouch cancer.^13^ Since then, advances in medical therapies as well as endoscopic diagnostic and therapeutic modalities have reshaped the management of IBD and ileal pouch care. This study focuses on confirmed pouch cancer in inflammatory bowel disease (IBD) patients, evaluating current management strategies, recurrence and progression, and mortality while providing insights into the ongoing research in this critical area.

## Patients and Methods

Patients were consecutively extracted from our prospectively maintained and institutional review board–approved Pouch Registry at Columbia University Irving Medical Center and New York Presbyterian Hospital between December 2019 and May 2025. A detailed review of electronic medical records was conducted for patients who had not withdrawn research authorization to complete clinical and diagnostic data collection.

### Inclusion and Exclusion Criteria

Patients aged 18 years or older who underwent IPAA for IBD complicated by confirmed cancer in the ileal pouch were included. Patients with precancerous conditions, including low-grade dysplasia (LGD), high-grade dysplasia (HGD), indefinite for dysplasia, or anal intraepithelial neoplasia (AIN), without a definitive cancer diagnosis, were excluded. Additionally, patients with IPAA performed for familial adenomatous polyposis or other colonic pathologies were considered beyond the scope of this study.

### Demographic and Clinical Variables

Demographic and clinicopathological variables included smoking status, excessive alcohol consumption, and significant comorbidities (eg, congestive heart failure, coronary artery disease, chronic obstructive pulmonary disease, renal insufficiency, nongastrointestinal cancer, cerebrovascular accident, and liver failure). Additionally, personal history of PSC, family history of IBD or CRC in first-degree relatives, prior diagnosis of pouch dysplasia and its management, and concurrent pouch disorders including chronic pouchitis, cuffitis, CD of the pouch, and evidence of mucosectomy were documented. The presence of synchronous neoplasia, defined as neoplasia occurring within 3 months of the current diagnosis, was also recorded.

### Pouch Cancer Characteristics

Pouch cancer locations included the anal canal, anal transition zone (ATZ), cuff, pouch body, or prepouch ileum. All available endoscopic evaluations of the pouch prior to the cancer diagnosis were assessed, and confirmatory pouchoscopy was performed within 3 months of diagnosis in cases where the tumor was suspected based on imaging. Relevant radiological imaging work-up was evaluated. Pathologic findings were confirmed by an expert gastrointestinal pathologist specializing in IBD. Therapeutic modalities, including endoscopic, surgical, and systemic treatments (eg, chemotherapy, radiotherapy, somatostatin analogs), were documented.

### Outcome Measurements

The primary outcomes included the recurrence or progression of pouch cancer and overall survival. The secondary outcomes encompass incidence, diagnosis, tumor location, histological type, tumor stage, and therapeutic approach.

### Statistical Analysis

Descriptive statistics were computed for all variables. This included mean and standard deviations or medians and interquartile ranges (IQRs) for continuous factors, and frequencies for categorical factors. Patient survival analysis was depicted using Kaplan–Meier curves. All statistical analyses were performed with the SPSS software version 29 (IBM, Chicago, IL).

## Results

A total of 10 patients (1.4%) with ileal pouch cancer were identified from the Pouch Registry, which included 699 patients. The incidence rate was 0.5 per 100 person-years. Baseline characteristics are summarized in Table 1.

**Table 1:** Demographic and Baseline Characteristics

Age at diagnosis of IBD, y + SD	21.6 + 9.3	

Median age at diagnosis of pouch cancer, y (IQR)	59.2 (43.7–63.8)	
Gender (%)	Female	5 (50)
Smoking (%)	Current	3 (30)
	Past	2 (20)
Excessive alcohol consumption (%)	2 (20)	
Comorbidities (%)	Heart failure or coronary artery disease	1 (10)
	Cerebrovascular accident	1 (10)
	Renal insufficiency	2 (20)
	Non-gastrointestinal cancer	2 (20)
	Liver failure	1 (10)
Family history of IBD (%)	2 (20)	
Family history of CRC	0	
PSC (%)	1 (10)	
Indication of colectomy (%)	Medically refractory disease	3 (30)
	CAN	7 (70)
Mucosectomy in total (%)	5 (50)	
Mucosectomy for CAN (%)	9 (90)	
Interval from pouch construction to cancer diagnosis, y (IQR)	11.2 (5.3–30.5)	
Routine endoscopy surveillance (%)	9 (90)	
Complications of IPAA		
Chronic pouchitis (%)	5 (50)	
CD of the pouch (%)	2 (20)	
Chronic cuffitis (%)	1 (10)	
History of postcolectomy neoplasia prior to inception (%)	4 (40)	
Presacral sinus (%)	4 (40)	
Treatment for pouchitis (%)		
Antibiotics	4 (40)	
Budesonide	1 (10)	
Vedolizumab	2 (20)	
Ustekinumab	1 (10)	

SD, standard deviation.

### Demographic and Clinical Features

The primary indication for proctocolectomy with IPAA was CAN (n = 7, 70%), followed by medically refractory disease (n = 3, 30%). The median interval from pouch construction to cancer diagnosis was 11.2 years (IQR 5.3–30.5). Among them, 5 presented with chronic pouchitis, 1 with cuffitis, 2 with CD of the pouch, and 4 with a presacral sinus, which were treated with endoscopic sinusotomy. Mucosectomy was performed in 5 patients.

**Figure 1:**
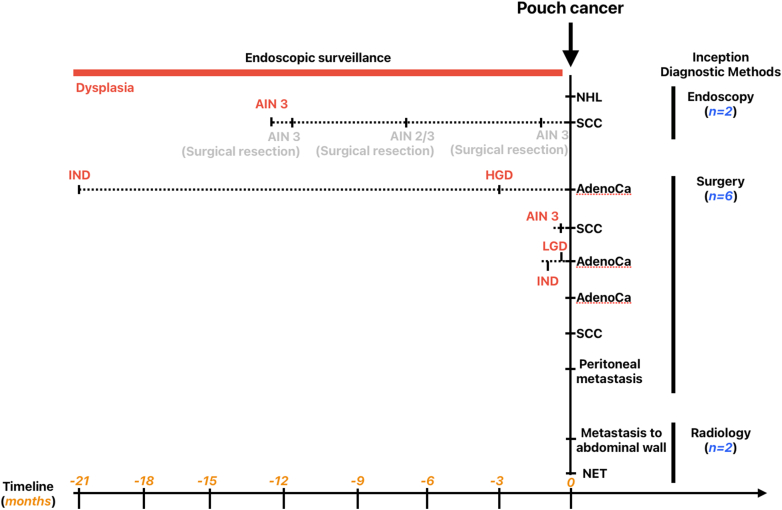
Inception diagnosis of pouch cancer and precancerous lesions during surveillance. AIN, anal intraepithelial neoplasia; IND, indefinite for dysplasia; NHL, non-Hodgkin's lymphoma.

Nine of 10 (90%) patients underwent routine annual endoscopic examinations for disease monitoring or cancer surveillance before the index diagnosis of cancer. Image-enhanced endoscopy (IEE), ie narrow-band imaging or dye-based chromoendoscopy, were performed at least once in 7 patients (70%) to asses mucosal surface and vascular pattern during surveillance. Four patients (40%) were diagnosed with pouch dysplasia before their cancer diagnosis, including one with HGD, 2 with AIN 3, and 1 with indefinite for dysplasia and LGD. Two underwent (50%) IEE and 3 had a history of CAN before pouch construction. Three experienced a diagnosis change from dysplasia to cancer within 3 months, confirmed by evaluation of surgical pathology (Figure 1).

### Endoscopic and Histopathological Features

**Figure 2:**
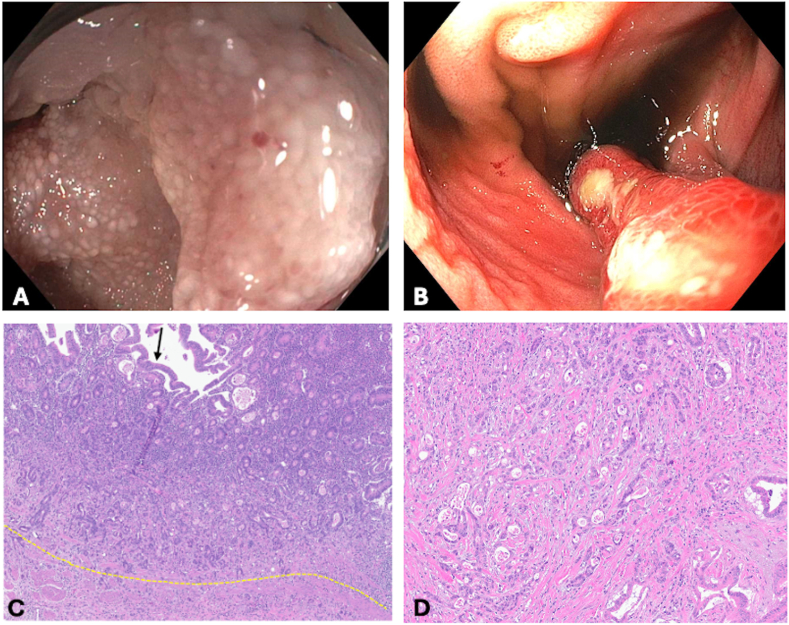
Adenocarcinoma. (A) Endoscopic evaluation of patient 1: Prepouch ileum stricture with normal-appearing mucosa resulting in adenocarcinoma after resection. (B) Endoscopic evaluation of patient 2: Pouch body fold with erythematous mucosa and ulcerated surface. (C) Histological section of patient 1: Invasive moderately to poorly differentiated adenocarcinoma invading the mucosa and submucosa (yellow line traces the muscularis mucosae). The overlying surface epithelium shows low-grade dysplasia (black arrow). H&E-stain, 100x magnification. (D). Histological section of patient 1: A higher power view showing adenocarcinoma infiltrating through the muscularis propria of the small intestine, which led to stricture formation clinically. Hematoxylin and eosin-stain, 200x magnification.

The inception diagnostic methods were endoscopic evaluation (n = 2, 20%) and surgical resection (n = 6, 60%), followed by cross-sectional imaging (n = 2, 20%). Lesions were located in the cuff (n = 3, 30%), ATZ (n = 3, 30%), pouch body (n = 2, 20%), and prepouch ileum (n = 1,10%; Figure 2). Of those, one had synchronous lesions in both ATZ and the pouch body. The endoscopic appearance varied, including stricture formation, diffuse inflammation with ulceration, and mass-like features (see Table 2). Notably, no polypoid lesions were observed. Among 2 patients with distant metastasis in whom pathology findings suggested a colonic origin, neither visible lesions on pouchoscopy nor neoplastic changes in random biopsies were detected (See Table 2).

**Table 2:** Endoscopic Features, Histological Findings, and Oncological Outcomes

Diagnosis methods (%)	
Endoscopy	2 (20)
Radiology (pasitron emission tomography/computed tomography, computed tomography–guided biopsy)	2 (20)
Surgical resection	6 (60)
IEE	
Surveillance	7 (70)
Precancerous lesions (n = 4)	2 (50)
Location of cancer (%)	
ATZ/Anal canal	3 (30)
Cuff	3 (30)
Pouch body	2 (20)
Small bowel (prepouch ileum)	1 (10)
Distant metastasis	2 (20)
Ascites liquid	1 (10)
Abdominal wall	1 (10)
Endoscopic features, within 3 mo of cancer diagnosis (%)	
Polypoid	0
Nonpolypoid	
Normal	2 (20)
Mass	2 (20)
Stricture	3 (30)
Inflammation/Ulcer	3 (30)
Histology (%)	
Adenocarcinoma	5 (50)
SCC	3 (30)
Others (NET and non-Hodgkin lymphoma)	2 (20)
Cancer staging at diagnosis (%) • Adenocarcinoma (5) • SCC (3) • NET (1) • Non-Hodgkin lymphoma (1)	
Stage I	3 (30)
Stage IV	2 (20)
Stage IIA	3 (30)
Stage IV	1 (10)
Stage I	1 (20)
Management (%)	
Endoscopic resection	0
Surgical intervention	5 (50)
Transanal excision	2/5 (40)
Pouch conversion from J to K	1/5 (20)
Small bowel resection	1/5 (20)
Chemotherapy or radiation therapy	6 (60)
Radical therapy	2/6 (33.3)
Adjuvant treatment for surgery	2/6 (33.3)
Palliative therapy for metastatic disease	2/6 (33.3)
Other (Lancreotide)	1 (10)
Recurrence/progression (%)	3 (30)
Mortality (%)	1 (10)
Median duration of follow-up, y (IQR)	2.8 (1.5–5.2)

**Figure 3:**
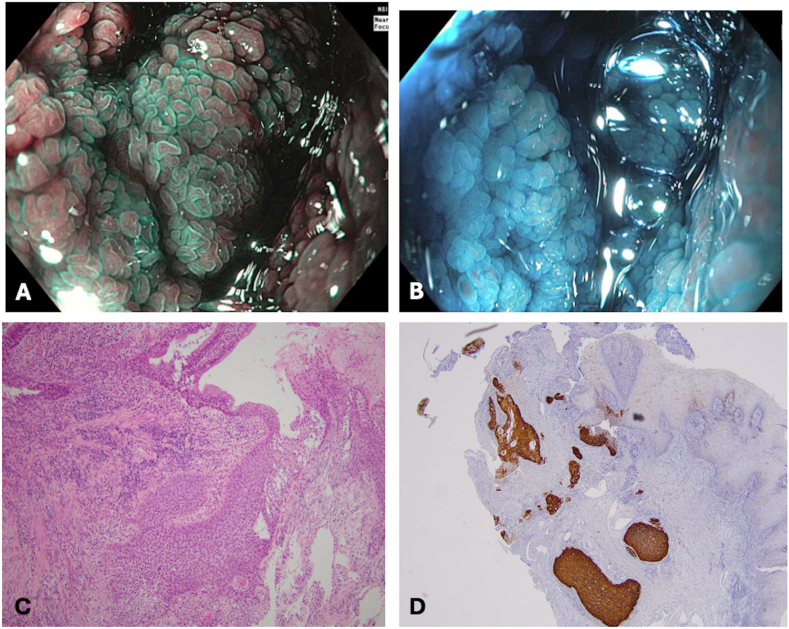
Invasive SCC at a strictured cuff. (A) Endoscopic evaluation with narrow band-imaging and magnification, showing an ill-defined nodular area. (B) Endoscopic evaluation with dye-chromoendoscopy and magnification did not demonstrate any distinct zones of hypo or hyperstaining. (C) Histological section: SCC arising in the squamocolumnar junction. H&E-stained section, 100x magnification. (D) Immunohistochemical stain for p16 shows strong expression in the invasive carcinoma at the squamocolumnar junction, suggesting an association with HPV. H&E-stained section, 40x magnification.

**Figure 4:**
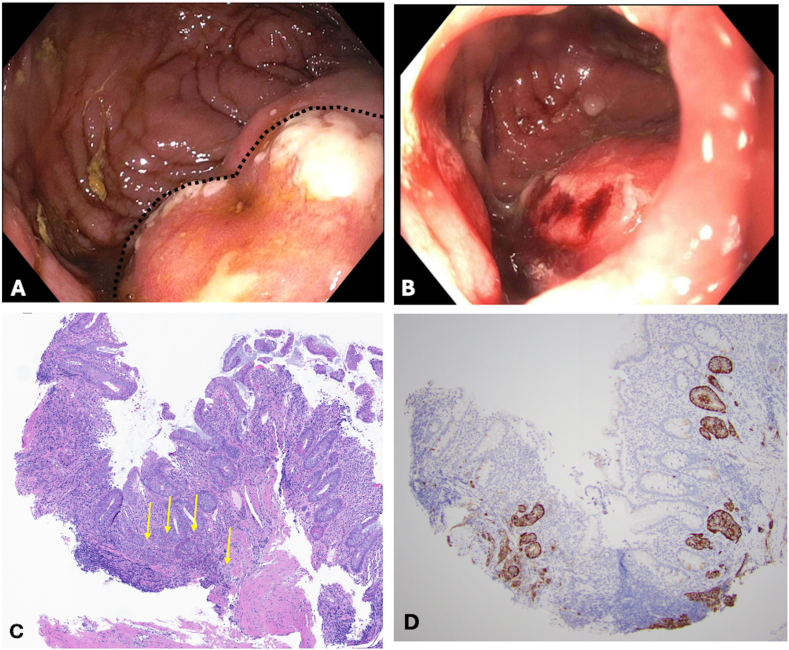
Well-differentiated NET in the distal pouch. (A) Endoscopic visualization: a mass-like formation (outlined) covered by whitish fibrin is observed in the anterior distal pouch. (B) Endoscopic visualization: Tumor with friable mucosa and oozing blood following biopsies. (C) Histological section: Small intestinal mucosa with nests of tumor cells at the base of the mucosa and infiltrating into muscularis mucosae (arrows). H&E-stain, 40x magnification. (D) Histological section: Synaptophysin immunohistochemical stain highlights NET. 40x magnification.

Adenocarcinoma was identified in 5 patients (50%), with 1 case exhibiting concomitant mucinous and signet ring features involving squamous mucosa. Squamous cell carcinoma (SCC) was diagnosed in 3 patients (30%), each presenting with distinct tumor locations and varying disease courses. The first case with SCC, a female patient with a lesion localized in the ATZ and positive for high-risk human papillomavirus (HPV) in situ hybridization, had persistent disease despite undergoing surgical resection. The second case involves a male patient with SCC of the rectal cuff (Figure 3), who evolved favorably following transanal excision. The tumor showed overexpression of p16, although HPV tested negative. The third case was a female patient with SCC in the ATZ, who received chemoradiotherapy (CRT) with curative intent and has shown no signs of recurrence during follow-up. Other rare histological types included a neuroendocrine tumor (NET) in the pouch body (Figure 4) and a diffuse large B-cell lymphoma (non-Hodgkin lymphoma) in the cuff.

Five patients required surgical intervention with curative intent. Among these, 2 had transanal excision, one underwent conversion from J pouch to K pouch, 1 required a small bowel resection, and 1 is pending surgery. Six patients received chemotherapy or radiation therapy, including 2 as radical therapy, 2 as adjuvant treatment for surgical margin <1 mm, and the other 2 as palliative treatment for disseminated disease. The patient with a NET was treated with lanreotide and maintained stable disease. She also had a prior history of thyroid cancer resection and marginal zone lymphoma treated with rituximab, both without evidence of recurrence. None of the patients received endoscopic resection therapy.

### Outcomes

**Figure 5:**
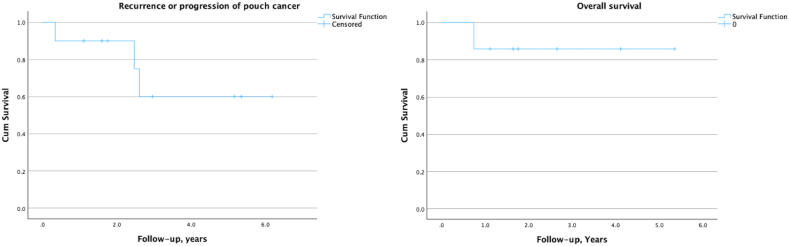
Survival curves for recurrence/progression and overall mortality in pouch cancer.

Recurrence or progression of cancer occurred in three patients with a mean time to progression of 10.5 months (Figure 5), over a median follow-up period of 2.8 years (IQR: 1.5-5.2). One patient with adenocarcinoma in the prepouch ileum, who underwent small bowel resection, developed local recurrence 2.5 years later. The index surgical margins were negative for carcinoma but ambiguous for focal LGD due to suboptimal specimen orientation. The patient developed prepouch and inlet strictures with partial obstruction, requiring endoscopic balloon dilation and stricturotomy every 6 months. Surveillance biopsies were taken in all procedures, being negative for dysplasia until the most recent one. Focal cytological atypia—reduced goblet cells and epithelial dysmaturation-was found on a background of chronic enteritis, interpreted as ID, probably positive. The immunostain of p53 was wild-type. CT imaging showed no signs of malignancy. Due to prior history, surgical resection and end-to-end anastomosis were performed, revealing moderately to poorly differentiated adenocarcinoma at the prior anastomosis site. Another patient, who had undergone IPAA for locally advanced colonic adenocarcinoma with lymph node involvement at the time of pouch construction, received concomitant chemotherapy. Unfortunately, the disease progressed and metastasized to the peritoneum in 1.5 years, and the patient ultimately died. The third patient with SCC in the ATZ, pouch excision and conversion to a K-pouch were performed after 3 surgical excisions for high-grade squamous intraepithelial lesions and topical chemotherapy with 5-fluorouracil. Systemic CRT was administered due to surgical margins <1 mm, related to cauterization defects. However, after the 2-year follow-up, disseminated disease was detected in the small bowel, and the patient is currently undergoing chemotherapy. (Table 2). All patients survived during follow-up except 1, resulting in a 5-year cumulative survival of 88.9% (Figure 5).

## Discussion

Pouch cancer in patients with IBD, though rare, remains one of the most feared complications due to the associated comorbidities, often leading to pouch excision, and the potential for significant mortality. Despite the previous efforts to explore the incidence and the risk factors related to pouch cancer, the natural history and the management of the disease are not well defined.

The prevalence of pouch cancer varies across studies but remains generally low. Among large registries, the Cleveland Clinic reported 15 cases (0.5%) in a cohort of 3203 patients from 1984 to 2009, with cumulative incidence rates of 0.2%, 0.4%,0.8%, 2.4%, and 3.4%, at 5, 10, 15, 20, and 25 years postpouch construction, respectively.^4^ In a subsequent cohort (2010-2020), 6 patients (0.16%) developed invasive adenocarcinoma out of 3672 patients.^8^ Most recently, the Mayo Clinic identified 2 cases (0.15%) among 1319 patients.^14^ In our cohort, 10 out of 699 patients (1.4%) developed pouch cancer over 5 years, suggesting a slightly increased incidence compared to the latest reports. However, the small number of events limits the precision of the incidence estimate.

Several risk factors have been proposed for pouch neoplasia, with precolectomy CAN being the only well-established one.^4^^,^^10^ Current guidelines recommend annual pouchoscopy for patients in this high-risk group.^15^, ^16^, ^17^ In our study, 7 of 10 patients underwent TPC-IPAA for CAN, and 3 of them received mucosectomy. This is in line with previous research suggesting that mucosectomy does not eradicate the risk of recurrence at the ATZ,^4^^,^^8^^,^^9^^,^^13^^,^^18^ likely due to the difficulty in removing all rectal columnar mucosa. Worth noting, traditional risk factors, such as PSC or a family history of CRC, were infrequent in our patients.

Regarding precancerous conditions, the significance and management of LGD remain controversial. Distinguishing true LGD from inflammation due to chronic pouchitis and cuffitis can be difficult. Urquhart et al. reported that half of the patients initially diagnosed with LGD showed resolution of neoplasia on follow-up pouchoscopy,^2^ while a higher resolution rate of 72.7% was observed by the Cleveland Clinic.^19^ This suggests that LGD may be stratified into high-risk (persistent or progressive LGD) and low-risk (no subsequent LGD) groups. However, close endoscopic surveillance is warranted, as the progression rate from LGD to advanced lesions including HGD and CRC, has been reported at approximately 25.0% in ileal pouch patients, and 5%–20% in UC patients without TPC-IPAA, over a median follow-up of 4 years.^10^^,^^20^, ^21^, ^22^ In our cohort, one patient initially diagnosed with ID progressed to HGD within 1.5 years. Additionally, 3 patients presented with HGD at the time of initial diagnosis, bringing the total to 4, had lesions upgraded to cancer within 3 months following repeat endoscopy or surgical sampling. The short interval between dysplasia and cancer diagnoses suggests sampling limitations, rather than true tumor progression.

Diagnosing pouch cancer is challenging due to the heterogeneous nature and vertical growth pattern of IBD-associated neoplasia, which limits adequate tissue sampling from deeper layers via endoscopy, even with advanced techniques such as biopsy-over-biopsy or tunneling biopsy. Endoscopic features of pouch malignancy may be nonspecific and atypical, such as ulcerations, deforming masses, or strictures.^4^ We observed that 20% of patients had normal-appearing mucosa, 30% had inflammation or ulceration, 30% had strictures, and 20% presented with mass-like features. Targeted biopsies (from masses and strictures) and random biopsies (from normal mucosa) are performed to enhance diagnostic yield. IEE, as recommended for surveillance by guidelines,^23^ was performed at least once in 70% of patients during endoscopic surveillance. Notably, while endoscopy detected precancerous lesions in 4 patients (40%), it identified cancer in only two. Five patients (50%) required either surgical resection or radiology to establish the diagnosis at inception. This observation aligns with previous studies, which showed that most diagnoses of pouch cancer were made from surgical specimens.^24^

Histologically, adenocarcinoma constituted the predominant type in previous reports,^13^ which aligns with our findings. Adenocarcinoma in the cuff or pouch appeared to share histomorphological and molecular features with ulcerative colitis-associated adenocarcinoma rather than small bowel adenocarcinoma.^24^ SCC in patients who underwent IPAA is rare, with only 9 cases reported in the literature.^25^^,^^26^ However, we observed a higher proportion of SCC in our cohort (n = 3, 30%), compared to 6.67% in a prior study.^19^ Two cases of the SCC were identified in ATZ and one in the cuff (rectal stump). SCC originating from the columnar mucosa in the colon or rectum is uncommon.^27^ Unlike anal cancer, which is strongly associated with HPV infection, particularly types 16 and 18, the role of HPV in CRC remains unclear.^20^ Most of them were reported in the setting of chronic inflammation, such as IBD and prior radiation exposure.^25^ While the association between pouch SCC and cervical neoplasia remains unclear, in our study, 2 of the 3 patients with SCC were female and tested negative for Papanicolaou smear. Other histological types as non-Hodgkin lymphoma^28^^,^^29^ and NETs,^21^^,^^30^ although infrequent, have been documented in case reports. The most common locations of pouch cancer are the cuff and ATZ,^13^^,^^22^ which is consistent with our results. It is noteworthy that metastatic disease of colonic origin developed in 2 patients without any primary endoscopic abnormalities in the pouch.

The choice of therapeutic modalities, though not well-defined, is often based on the location of the cancer and the histology. For pouch adenocarcinoma, treatment typically involves abdominoperineal resection with end ileostomy or continent ileostomy (K-pouch) when the disease is localized.^13^ CRT may be considered in cases of disseminated disease. The management of SCC in the ileal pouch is less clear. While SCC in the pouch body and cuff likely requires pouch excision, those arising in the anal canal below the dentate line may be treated with radical CRT, potentially preserving the pouch. Nonetheless, concerns exist regarding the risk of radiation-induced pouchitis in patients with IPAA. There is one documented case of SCC at the ATZ successfully treated with CRT with a complete response and no adverse effect on pouch function.^26^

Endoscopic mucosal resection and endoscopic submucosal resection are emerging, less invasive options for early-stage CRC. While colectomy was traditionally the preferred treatment for CAN, endoscopic mucosal resection, and endoscopic submucosal resection are now considered viable alternatives for managing visible and resectable lesions.^31^^,^^32^ However, lesions with a bottom-up growth pattern may spare the surface epithelium, appearing flat or invisible,^33^ which limits their suitability for endoscopic resection. Notably, none of the patients presented polypoid resectable lesions, raising questions about the applicability of these techniques in pouch patients.

Pouch cancers are traditionally associated with a poor prognosis.^4^^,^^24^ A study by the Cleveland Clinic, with a median follow-up of 2 years, reported a recurrence or progression rate of 45.5% in 11 patients who initially underwent curative resection of the tumor, and a mortality rate of 42.9%, resulting in a 5-year cumulative overall survival rate of 49.5%.^13^ In our cohort, a higher proportion of patients with precolectomy CAN was observed than in the Cleveland Clinic series (70% vs 50%).^19^ Nevertheless, 70% of our patients were diagnosed at a localized stage of the tumor and underwent surgical resection or received CRT with curative intent. Over a median follow-up period of 2.9 years (IQR 1.5–5.2), recurrence or progression occurred in 30% of cases, and mortality was 10%, yielding a markedly improved 5-year cumulative survival of 88.9%.

This case series provides valuable insight into the natural history and management of pouch cancer. In patients with risk factors, endoscopic surveillance with biopsies may be insufficient for the definitive diagnosis of cancer, requiring radiological workup and surgical sampling. The diagnosis upgrade from precancerous lesions to cancer following repeat endoscopy or surgical resection within 3 months highlights the need for caution in clinical decision-making, as residual neoplastic tissue is a major contributor to cancer progression and poor prognosis. The higher incidence of ileal pouch SCC observed in this cohort suggests that a detailed evaluation of the cuff and ATZ should be performed. A key strength of our study is that all patients underwent endoscopic evaluation before or after the diagnosis of cancer, whether the primary lesion was in the pouch or manifested as distant metastasis. Furthermore, all biopsies were reviewed by a consistent group of expert gastrointestinal pathologists, uniformly trained in interpreting IBD-associated histological activity and neoplasia.

However, the main limitation of this study is its retrospective nature and small sample size, which precluded the performance of univariate and multivariate analyses. The low event rate for individual cancer subtypes further limits more granular analysis. As is well-known, sporadic colonic adenocarcinoma and anal SCC per se present distinct prognoses, with SCC generally associated with a lower overall survival rate. When assessing the oncological outcomes of pouch cancers, direct comparison was limited due to the higher proportion of SCC observed in our group compared to previous reports. Another limitation is that this study was conducted at tertiary referral centers, potentially introducing selection bias. Lastly, there is potential for reporting bias, as it is possible that patients with an ileal pouch may not have participated in routine surveillance pouchoscopy. Nevertheless, our findings provide a foundation for future studies, particularly in identifying risk factors for the development of SCC and alternative diagnostic strategies, such as regimented HPV in situ hybridization in cuff/ATZ tissue, for SCC in the ileal pouch. Further studies are warranted to elucidate the resectability of colitis-associated dysplasia in the ileal pouches and the need for upgraded sampling techniques in decision-making for management.

## Conclusion

This case series evaluates 10 ileal pouch cancers extracted from 699 patients who underwent ileal pouch construction for IBD. While adenocarcinoma remained the most frequent cancer, nonadenocarcinoma accounted for 50% of the cases, with a higher incidence of SCC at ATZ/cuff, highlighting the importance of this often-neglected area. Endoscopy was helpful in diagnosing precancerous lesions, but not adequate for the definite diagnosis of cancer, which frequently required surgical sampling and radiological evaluation. More than half of the patients were diagnosed at a localized stage, allowing treatment with curative intent. Over a median follow-up of 2.8 years, recurrence or progression occurred in 30% of patients, and mortality was 10%, resulting in an improved 5-year cumulative survival of 88.9%. While the necessity of close endoscopic surveillance has been debated, early-stage diagnosis of pouch cancer may positively impact oncological outcomes.
